# Expression of Random Sequences and de novo Evolved Genes From the Mouse in Human Cells Reveals Functional Diversity and Specificity

**DOI:** 10.1093/gbe/evae175

**Published:** 2024-12-12

**Authors:** Silvia Aldrovandi, Johana Fajardo Castro, Kristian Ullrich, Amir Karger, Victor Luria, Diethard Tautz

**Affiliations:** Max-Planck Institute for Evolutionary Biology, Dept. Evol. Genetics, Plön 24306, Germany; RG Development & Disease, Max Planck Institute for Molecular Genetics, Berlin 14195, Germany; Max-Planck Institute for Evolutionary Biology, Dept. Evol. Genetics, Plön 24306, Germany; Science and Technology Academy, University of Kiel, Kiel 24118, Germany; Max-Planck Institute for Evolutionary Biology, Dept. Evol. Genetics, Plön 24306, Germany; IT-Research Computing, Harvard Medical School, Boston, MA 02115, USA; Department of Neuroscience, Yale School of Medicine, New Haven, CT 06510, USA; Division of Genetics and Genomics, Boston Children's Hospital, Harvard Medical School, Boston, MA 02115, USA; Department of Systems Biology, Harvard Medical School, Boston, MA 02115, USA; Max-Planck Institute for Evolutionary Biology, Dept. Evol. Genetics, Plön 24306, Germany

**Keywords:** random peptides, de novo evolved genes, differential growth, transcriptomic analysis

## Abstract

Proteins that emerge de novo from noncoding DNA could negatively or positively influence cellular physiology in the sense of providing a possible adaptive advantage. Here, we employ two approaches to study such effects in a human cell line by expressing random sequences and mouse de novo genes that lack homologs in the human genome. We show that both approaches lead to differential growth effects of the cell clones dependent on the sequences they express. For the random sequences, 53% of the clones decreased in frequency, and about 8% increased in frequency in a joint growth experiment. Of the 14 mouse de novo genes tested in a similar joint growth experiment, 10 decreased, and 3 increased in frequency. When individually analysed, each mouse de novo gene triggers a unique transcriptomic response in the human cells, indicating mostly specific rather than generalized effects. Structural analysis of the de novo gene open reading frames (ORFs) reveals a range of intrinsic disorder scores and/or foldability into alpha-helices or beta sheets, but these do not correlate with their effects on the growth of the cells. Our results indicate that de novo evolved ORFs could easily become integrated into cellular regulatory pathways, since most interact with components of these pathways and could therefore become directly subject to positive selection if the general conditions allow this.

**Significance**

The study is a model experiment for understanding the possible impact of de novo gene emergence in mammalian cells. It shows that the majority of more or less random sequences expressed in such cells can influence the growth of the cells and could, therefore, become relevant for evolutionary adaptations.

## Introduction

New genes can evolve through duplication and divergence from existing genes, or de novo out of noncoding sequences (Tautz and Domazet-Lošo 2011; McLysaght and Guerzoni 2015; Van Oss and Carvunis 2019). While de novo evolution of genes was contentious for some time (Tautz 2014), it is now supported by ample evidence, including functional studies (Cai et al. 2008; Li et al. 2010; Fellner et al. 2015; Gubala et al. 2017; Xie et al. 2019; Vakirlis et al. 2020; Xie et al. 2020; Lange et al. 2021; Rivard et al. 2021; An et al. 2023; Qi et al. 2023), and inferences drawn from expressing random coding sequences (Digianantonio and Hecht 2016; Bao et al. 2017; Neme et al. 2017; Tretyachenko et al. 2017; Knopp et al. 2019; Bhave and Tautz 2021; Castro and Tautz 2021; Knopp et al. 2021; Babina et al. 2023; Frumkin and Laub 2023).

Comparative analyses of genome and transcriptome sequences have revealed that every evolutionary lineage expresses a large number of lineage-specific transcripts with the potential to be converted into functional protein-coding genes (Carvunis et al. 2012; Neme and Tautz 2016; Durand et al. 2019; Zhang et al. 2019; Heames et al. 2020; Ruiz-Orera et al. 2020). However, functional analysis of de novo evolved genes remains a challenge. Apart of very general sequence features, such as transmembrane domains (Vakirlis et al. 2020), they harbor almost no sequence-based clues that would allow us to draw inferences on the molecular and regulatory networks, they are part of. For mice, we have shown that deep transcriptome analysis of knockout versions of de novo evolved genes can still provide clues on possible functions (Xie et al. 2020). An alternative to knockout approaches is overexpression of such genes and to study their fitness consequences for the respective cells (Vakirlis et al. 2020).

We have chosen here a combination of previous approaches to study de novo evolved genes in a human cell line. First, we show that the expression of a library of random sequences leads to differential growth effects in the cells that harbor different random peptides. In a second experiment, we ask whether genes that evolved de novo in the mouse lineage have specific effects in human cells when over-expressed. This approach addresses the question of whether a de novo evolved protein can interact with a molecular network in a species in which it did not evolve and could, therefore, not have been adapted for function in this cellular context. This is akin to expressing random sequences but pre-selected for sequence variants that were already evolutionarily tested in another lineage (Heames et al. 2023).

We show that both random peptides and de novo genes represent a range of structural protein features, which do not, however, correlate with their growth effects on cells or the transcriptomic responses that they elicit in the cells. Hence, the current structural predictions alone are not sufficient to predict possible functional effects of random ORFs in eukaryotic cells. Nevertheless, since most clones elicit specific effects in the cells, we conclude that it should be easy for a de novo evolved gene to become integrated into cellular pathways, if the cellular, organismal, or environmental selection pressures allow this.

## Results

We used the human Flp-In™ T-REx™ 293 cell line (FITR293) for all expression experiments. This cell line permits the generation of stable clones where the construct is integrated into a specific target site in the genome, from which it can be induced to be expressed. We used this on the one hand to express random sequences and on the other hand to express cDNAs from mouse de novo genes (Mdngs).

### Random Sequence Expression

The concept of expressing random sequences as a model for de novo evolution is analogous to an experiment that we had done before in Escherichia *coli cells* (Neme et al. 2017), but here we use eukaryotic-specific genomic features in the flanking regions. Specifically, we used a Kozak sequence to facilitate translation, shorter leading sequences on the 5′-end of the random sequence, and codon-optimized flanking sequences on both sides of the random sequence using frequently used codons in the human genome (supplementary file S1, Supplementary Material online, supplementary fig. S1.1, Supplementary Material online).

For the generation of the random sequence library, oligonucleotides with the random sequences were first inserted in the multiple-cloning site of a bacterial plasmid followed by insertion into the FITR293 cell line through site-directed recombination (supplementary file S1, Supplementary Material online, supplementary figs. S1.1 and S1.2, Supplementary Material online). The resulting cells were morphologically indistinguishable from the parental cell line and they had approximately the same doubling time, judging from the time required between passages.

Each cell in the library is thus modified to express a single 174-nucleotide-long inserted sequence with 150 random nucleotides flanked by two constant codons at the 5′ end, and a 6-histidine tag at the 3′ end (supplementary file S1, Supplementary Material online, supplementary fig. S1.1, Supplementary Material online). This inserted sequence is driven by a CMV promotor, its non-translated 3´-end plus polyadenylation signal is derived from the bovine growth hormone (bGH) (supplementary file S1, Supplementary Material online, supplementary fig. S1.2, Supplementary Material online). Each random sequence acts as a barcode, and this makes it possible to quantify the relative number of cells in the cell population expressing it using an amplicon sequencing approach. In this way, one can monitor how the proportion of individual sequences changes over time under joint growth conditions.

The starting library was sequenced using Illumina MiSeq. We found 3,708 different clones with a sufficiently high representation to conduct statistical analysis (supplementary table S1, Supplementary Material online). The distribution of predicted peptide lengths in the library matches the expected distribution of a library of random sequences of this length (supplementary file S1, Supplementary Material online, supplementary fig. S1.3, Supplementary Material online). Other molecular features of the nucleotide and predicted peptide sequences also match the expected distributions of values for random sequences. For example, GC content of both the full-length reads and the corresponding predicted ORFs is narrowly distributed around 50%. When looking at the average frequency of each nucleotide at each position only in the random part of the sequence, there is a slight bias towards a higher content of thymine versus a lower content of adenine in all positions (supplementary file S1, Supplementary Material online, supplementary fig. S1.4, Supplementary Material online), but this does not much affect the overall features of the library. Intrinsic disorder or aggregation propensity distributions of the different random peptides depend more on the respective length of the encoded peptides (supplementary file S1, Supplementary Material online, supplementary figs. S1.5 and S1.6, Supplementary Material online).

### Competitive Growth of the Random Sequence Library

The library of random sequences was cultured on dishes with doxycycline in the medium to induce expression of the peptides over a period of 20 d, sampled and re-plated every two days before the cells reached confluence. They were bulk-sequenced at each of the 10 sampling steps to determine the proportion of cells containing each random ORF (supplementary table S2, Supplementary Material online).

To determine the overall growth trends for each clone, we used DESeq2 (Love et al. 2014) to assess whether a significant (*p*_adj_ < 0.05) change had occurred between time point 1 and time point 10. The corresponding log2-fold changes and *p*_adj_ values are listed for each clone in supplementary table S1, Supplementary Material online, together with the corresponding classifications. We found that 1,470 (40%) clones did not change significantly (group NS: Non-Significant), 1,934 (53%) clones declined in frequency (group DOWN), and 304 (8%) clones increased in frequency (group UP). In previous equivalent experiments in *E. coli,* we found that 27% to 65% were NS, 26% to 46% were DOWN, and 7% to 27% were UP clones (Neme et al. 2017; Castro and Tautz 2021). Hence, the percentages in the eukaryotic cells are generally within the same ranges, only the DOWN fraction seems to be a bit higher.

**Fig. 1.**
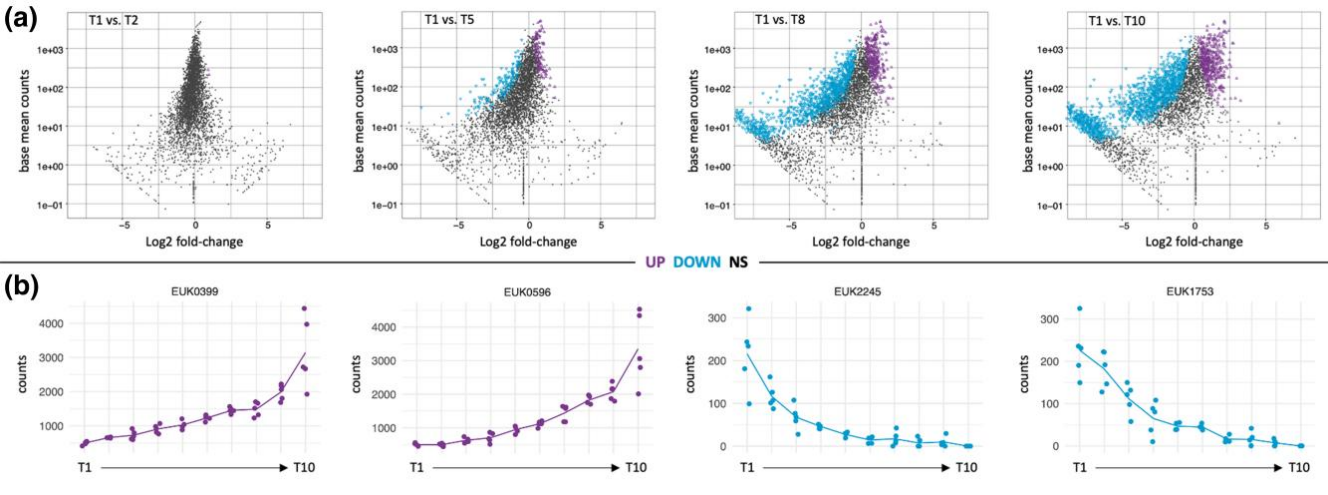
MA plots for all clones and single clone trajectories. a) Sum of all clone changes at four different time points of growth as base mean counts vs. log2-fold-change plots. Shown here are timepoints T2, T4, T8 and T10 compared to timepoint T1, combining all 5 replicates. Colored dots represent clones that are significant (*P*_adj_ < 0.05) in the DESeq2 analysis between the corresponding time points. b) Change trajectories for four clones across all 10 timepoints, two UP and two DOWN clones are shown. Replicates are represented as dots at each time point. Plots are based on the data provided in supplementary table S2, Supplementary Material online.

The progression in the change of frequency of clones across four different time points is displayed in Fig. 1a, where the mean base counts for each clone are plotted against the log2-fold change. As time and growth progress, fold changes increase in magnitude and become increasingly significant in the DESeq2 analysis (colored points), supporting the validity of the trends used for the group assignment. This is also evident from the example trajectories for four clones displayed in Fig. 1b.

**Fig. 2.**
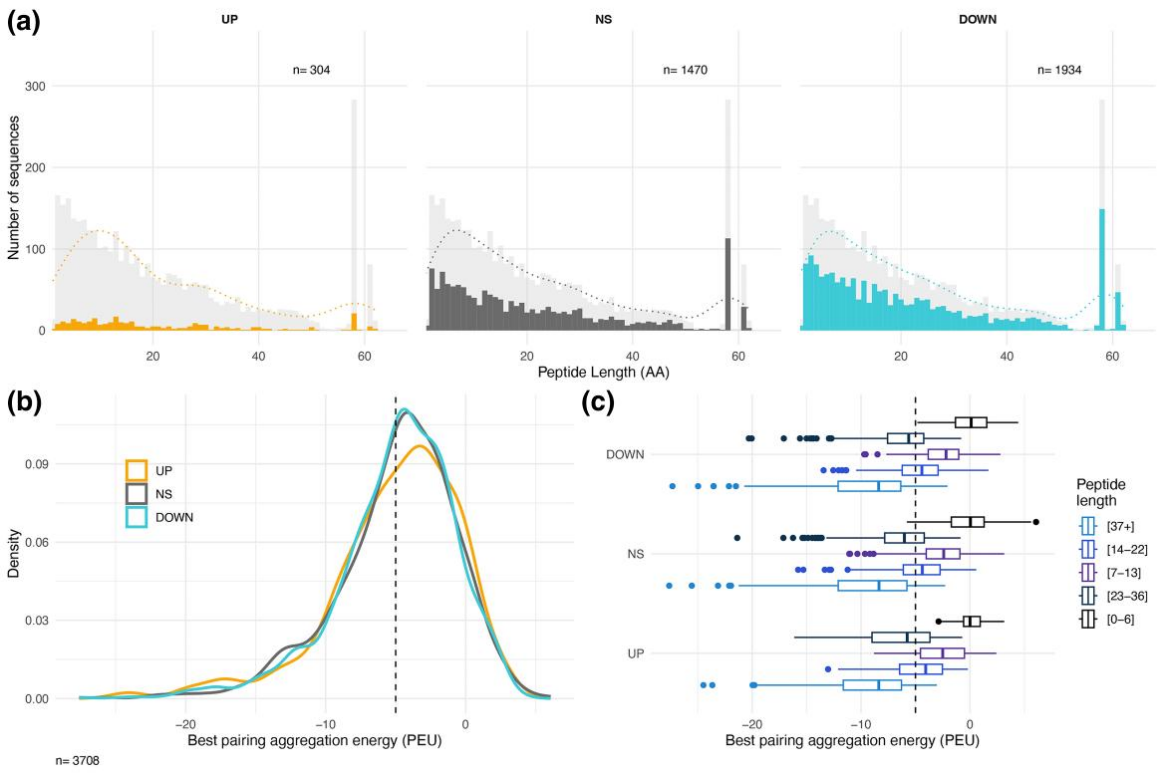
Features of sequences in each reaction group. a) Sequence length distribution in each group. Each panel shows the background distribution for all clones in the library as grey and the actual distribution for each of the classes in the respective colors (orange: peptides showing an increase in frequency (UP), dark grey: peptides showing no significant change in frequency (NS) and light blue: peptides showing a decrease in frequency (DOWN). Dashed lines represent the kernel density estimates for each group. b) Density plots for the best aggregation energy of sequences in each group, calculated by PASTA (PEU). Same color scheme as in a) for the three groups. c) Best aggregation energy of sequences in each group distributed according to length classes of the peptides. The lower and upper hinges correspond to the first and third quartiles (the 25th and 75th percentiles). The whiskers are the minimum and maximum data points up to 1.5 times the closest IQR. The data for all panels are included in supplementary table S1, Supplementary Material online. Note that the figure panels can be directly compared to results on random peptides expressed in *E. coli* (Figs. 2 and 6 in [Castro and Tautz 2021]).

Using the group assignments into UP, DOWN, and NS, we compared molecular features of the sequences in each group against the full database. Interestingly, unlike the clear correlation of length with group assignment observed in bacterial experiments (Castro and Tautz 2021), we do not observe such an effect for the random peptides expressed in the eukaryotic cell line (Fig. 2). The length distributions for each group are not significantly different from the distribution of the peptides in the whole library (Kolmogorov-Smirnov test, *P* >> 0.05) (Fig. 2a). Aggregation propensity was calculated with PASTA (Walsh et al. 2014), which calculates the free energy of predicted ß-strand intermolecular pairings for each sequence and provides the lowest value for each peptide as the best pairing. Lower aggregation energies mean that it is easier for the peptides to form amyloids or to aggregate. Aggregation energies lower than −5 pasta energy units (PEU) indicate possible amyloid formation. In a similar analysis in bacteria, we found that DOWN peptides tend to have lower PEUs, compared to the other two classes (Castro and Tautz 2021). This is not the case for the peptides that are expressed in the eukaryotic cell line, where the distributions of PEUs are not significantly different between the classes (Kolmogorov-Smirnov test, *P* >> 0.05) (Fig. 2b). This is also the case when one compares the different length classes for each group (Fig. 2c).

### Mouse de Novo Genes (Mdng)

To study the effects of the expression of de novo genes from the mouse in human cells, we chose a set of 16 mouse (*Mus musculus*) genes derived from a curated list of 119 candidate de novo genes in the mouse (Xie et al. 2019). This list was originally based on annotated transcripts with translated ORFs (confirmed by proteomic evidence) which can be found in the mouse reference genome, but not in distant mammals, including humans. The list of these genes with all relevant additional information is provided in supplementary table S3, Supplementary Material online. Eight of the genes are located in positions that are compatible with an origin from a bidirectional promoter, a mechanism that is thought to be often involved in the activation of new transcripts that can form de novo genes (Gotea et al. 2013; Jensen et al. 2013). Four of the genes were previously analyzed in knockout experiments in mice, Mdng2 and Mdng3 in (Xie et al. 2020), Mdng7 in (Xie et al. 2019) and Mdng15 in (Lu et al. 2019).

### Mdng Gene Evolutionary age and Protein Subcellular Localization

The detection of lineage-specific genes is evidently affected by the availability of genome data for close taxa. Given that the initial list of Mdng genes was drawn up at a time with more limited genome representations in the databases, we reassessed all of the genes in the study when they appeared in evolution, based on the latest genome releases. We found that most of the Mdng genes (13/16) are still only found in *Mus* species and thus likely appeared in the last two million years (supplementary table S3, Supplementary Material online).

The three remaining Mdng genes are slightly older, while still being specific to the rodent order. For Mdng12, a predicted protein sufficiently similar is present in the deer mouse *Peromyscus maniculatus (Pm)*. For Mdng14, similar predicted proteins are present in deer mouse and Roborovski's hamster (*Phodopus roborovskii (Pr))*, both belonging to the *Cricetidae* family that diverged ∼26.9 Mya from the *Muridae* family which includes *Mus musculus*. For Mdng15, similar predicted proteins are present in the shrew mouse *Mus pahari (Mp)*, a species within the *Muroidea* family, as well in the deer mouse (*Pm*), the Eurasian water vole *Arvicola amphibius (Aa)*, and the Bank vole *Myodes glareolus (Mg)*, all three *Cricetidae* family members (all alignments shown in supplementary file S2, Supplementary Material online). Thus, the Mdng12, Mdng14, and Mdng15 genes appeared before the divergence of *Muridae* and *Cricetidae* lineages with a split time estimated at ∼26.9 Mya (Kumar et al. 2022), i.e. they are not present in the human lineage.

Next, we evaluated the predicted subcellular localization of Mdng proteins by scoring the presence of signal peptides (SP) and transmembrane domains (TMD) (Kall et al. 2007; Almagro Armenteros et al. 2019; Bernhofer et al. 2021). TargetP SP scores are moderate (0.41—0.50) for 3 Mdng proteins (Mdng6, Mdng11, Mdng 13) and strong for a fourth (0.79, Mdng16). Additionally, two proteins appear to have a predicted TMD (Mdng3, residues 86 to 104; Mdng4, residues 79 to 111) while another, Mdng11, has two predicted TMDs (residues 20 to 38, 59 to 76). Thus, of the 16 Mdng proteins, three are predicted to be secreted (SP + TMD−, Mdng6, Mdng13, Mdng16), one likely is a transmembrane protein on the cell surface (SP + TMD+, Mdng11), and two others likely are transmembrane proteins (SP− TMD+, Mdng3, Mdng4) localized in membranes of intracellular organelles.

### Competitive Growth of Mdng

PCR products from cDNA copies of the Mdng genes discussed above were cloned into the same expression site in human FITR293 cells that were also used for expressing the random sequences. These clones were then used for further experiments.

**Fig. 3.**
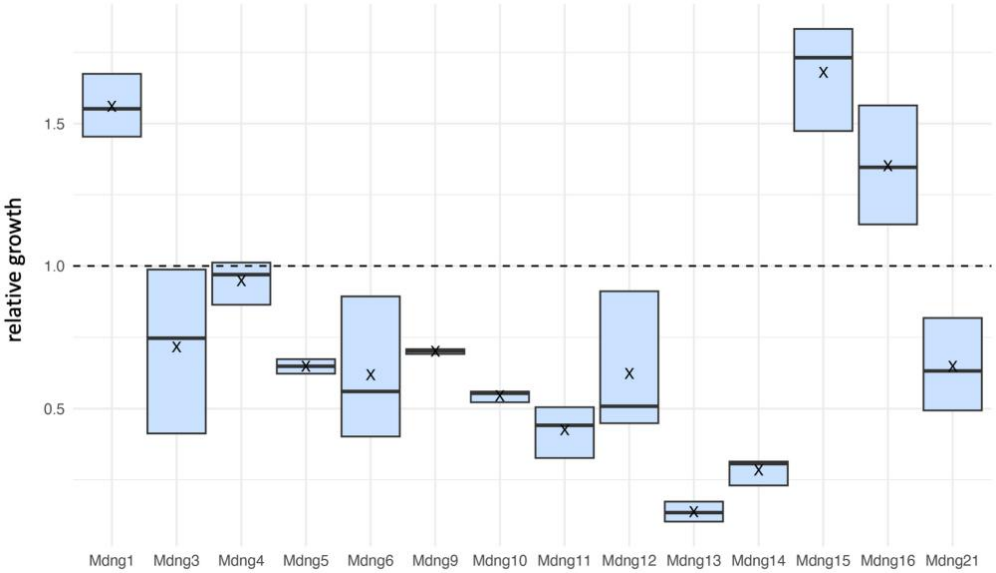
Growth differences of Mdng clones under common growth conditions. Relative growth differences are shown between the start and cycle 5. Boxes represent the three values for each clone, with averages marked as cross. Full data are in supplementary table S4, Supplementary Material online.

All clones showed normal growth characteristics under doxycycline induction conditions (i.e. when the mouse gene was expressed), indicating that this expression does not harm the cells in a major way. However, we then asked whether the expression of the mouse genes has a differential growth effect on the respective clones when grown under common conditions in the same flask. To test this, we combined samples from Mdng clones into a pool of cells and set up three replicates for growth across five successive cycles. Two clones (Mdng2 and Mdng7) failed to grow in this experiment, the other showed consistent growth trajectories across the cycles (supplementary table S4, Supplementary Material online). Three clones (Mdng1, Mdng15, and Mdng16) increased their cell numbers in the pool, while one (Mdng4) showed little relative change and the other clones declined to various degrees (Fig. 3).

### Transcriptomic Response Upon Mdng Expression

Genes evolved only in the mouse lineage can be considered as completely new genes in the genomic background of human cells. Hence, by expressing them in these cells, we simulate the first appearance of a de novo evolved protein-coding transcript that has not undergone further optimization in the respective cell background. By doing this for multiple independent genes, we ask whether their expression triggers a generic general response, or whether this is specific for each gene.

Interestingly, the expression of the individual Mdng RNAs varies substantially (Table 1), although they are all expressed from the same promotor and share the noncoding 3′-end of the RNA. When compared to the relatively constant expression of the hygromycin gene from the same vector construct, it varies between 2% and 94% of hygromycin RNA. This suggests that the mouse sequence part in the RNA could have either an effect on the CMV promotor strength or could affect the relative stability of the RNA. Interestingly, the two relatively highest expressed genes in the human cells (Mdng1 and Mdng15) are also the two genes with the highest average expression across mouse tissues (supplementary table S3, Supplementary Material online), indicating that their RNA may be particularly stable.

**Table 1:** Changes in human gene expression through expression of mouse de novo genes

	Mdng expression level in the experiment (normalized to hygromycin expression)	Number of human genes with > |2-fold| change and *P*_adj_ < 0.05	Number of GO terms *P* < 10^−3^	Top GO term with *P*-value and false discovery rate (FDR) *q*-value
Mdng1	0.94	300	4	GO:0061792 secretory granule maturation (*P* = 3.1E−04, FDR *q* = 1)
Mdng2	0.36	520	31	GO:0043269 regulation of ion transport (*P* = 1.2E−05, FDR *q* = 0.18)
Mdng3	0.39	1044	25	GO:0031620 regulation of fever generation (*P* = 3.7E−05, FDR *q* = 0.54)
Mdng4	0.04	77	1	GO:0010984 regulation of lipoprotein particle clearance (*P* = 5.2E−04, FRD *q* = 1)
Mdng5	0.25	266	14	GO:0007155 cell adhesion (*P* = 1.0E−0.5, FDR *q* = 0.15)
Mdng6	0.15	126	14	GO:0007155 cell adhesion (*P* = 2.0E−04, FDR *q* = 1)
Mdng7	0.52	35	0	none
Mdng9	0.16	188	41	GO:0003308 negative regulation of Wnt signaling pathway involved in heart development (*P* = 8.6E−06, FDR *q* = 0.12)
Mdng10	0.35	108	4	GO:0006700 C21-steroid hormone biosynthetic process (*P* = 4.7E−04, FDR *q* = 1)
Mdng11	0.02	405	55	GO:0007156 homophilic cell adhesion via plasma membrane adhesion molecules (*P* = 1.2E−14, FDR *q* = 1.2E−10)
Mdng12	0.25	118	15	GO:0042662 negative regulation of mesodermal cell fate specification (*P* = 1.4E−04, FDR *q* = 1)
Mdng13	0.07	1171	101	GO:0030198 extracellular matrix organization (*P* = 1.1E-09, FDR q = 1.6E-05)
Mdng14	0.17	744	37	GO:0071805 potassium ion transmembrane transport (*P* = 5.5E−06, FDR *q* = 0.08)
Mdng15	0.63	393	23	GO:0006873 cellular ion homeostasis (*P* = 7.0E-05, FDR q = 1)
Mdng16	0.15	2392	4	GO:0006700 C21-steroid hormone biosynthetic process (*P* = 1.3E−04, FDR *q* = 1)
Mdng21	0.07	1165	6	GO:0009097 isoleucine biosynthetic process (*P* = 8.8E−05, FDR *q* = 1)

The analysis of the transcriptomic response of the human genes in response to the expression of each mouse gene was assessed based on a DESeq2 (Love et al. 2014) analysis with a cutoff of *P*_adj_ < 0.05 and an at least 2-fold change of up or down expression.

The response to each of the mouse genes is unique, with a range between 35 and 2,392 significantly changed transcripts (Table 1). The weakest response was found for Mdng7, the strongest for Mdng16 (Table 1). The strength of the response is weakly, though not significantly, negatively correlated with the relative expression level of the mouse gene in the corresponding experiment (Spearman rho = −0.21, *P*(2-tailed) = 0.4).

GO terms for the human genes responding to the different Mdngs vary greatly, only Mdng7 shows no GO-term enrichment (Table 1). Also, the top enriched GO terms are different for each Mdng, only Mdng5 and Mdng6 show the same top term (cell adhesion) which is due to partially overlapping gene sets (including mucin 4, protocadherin 10, platelet endothelial aggregation receptor 1, claudin 7 and collagen 16A1, indicating that these two Mdngs may trigger somewhat similar overall responses in human cells. However, note that while the GO terms are associated with significant *P*-values, the false discovery rate (FDR) value *q* is mostly larger than 0.1 (Table 1). Still, we list them in Table 1, since they reflect nonetheless general tendencies in the human gene sets that are differentially expressed.

The most significant result is found for Mdng11 with the GO term “homophilic cell adhesion via plasma membrane adhesion molecules” (FDR *q* << 0.01). This is due to the upregulation of the genes and transcripts in the protocadherin alpha gene cluster on chromosome 5. Interestingly, Mdng11 has a predicted signal peptide and two predicted transmembrane domains and is thus likely to be located at the cell surface.

We also compared the human transcripts with the highest fold changes (the top 10 up and the top 10 downregulated, respectively). Interestingly, most (on average 14 out of 20 for each Mdng experiment) are human-specific transcripts without orthologues in the mouse (based on the gene annotation resources described in [Seal et al. 2023]) (supplementary table S5, Supplementary Material online). These include predicted coding genes with introns (i.e. candidates for de novo evolved genes), lncRNAs, as well as antisense transcripts, and fused transcripts between neighboring genes.

**Fig. 4.**
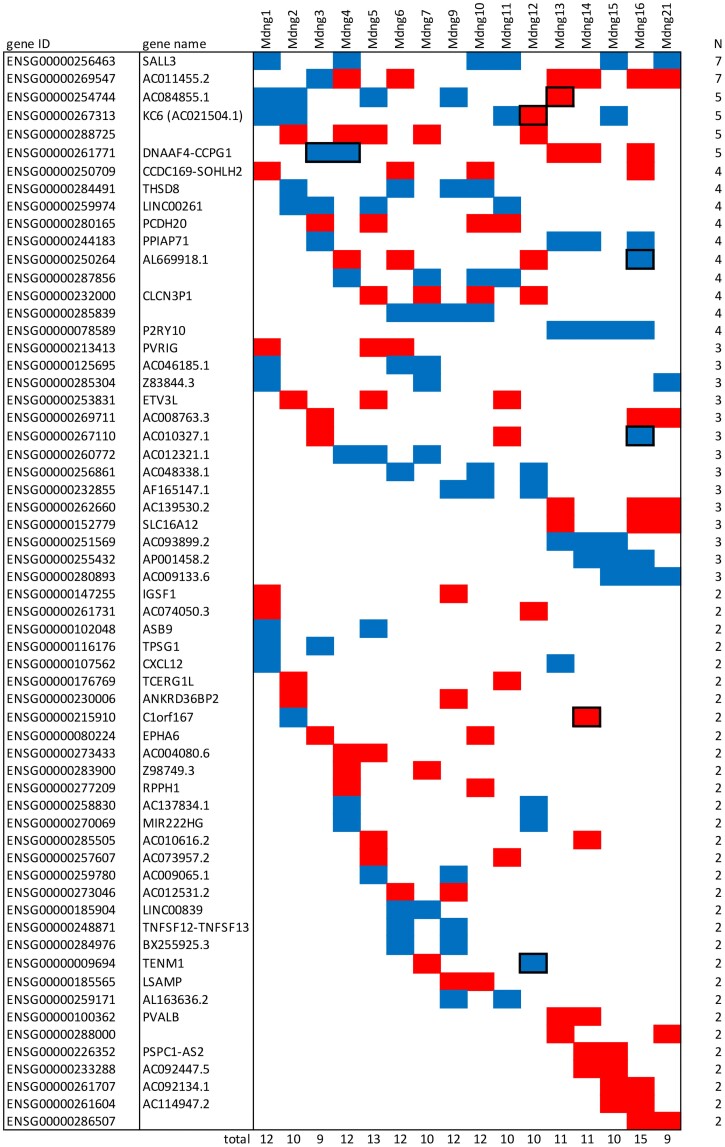
Matrix of genes that are differentially regulated in more than one of the top 20 gene sets for each Mdng. Blue boxes represent down-regulation, red boxes represent up-regulation (see supplementary table S5, Supplementary Material online for values). Outlined rectangles represent cases with opposite direction changes in different sets. The list is ordered with respect to the number (N) of recurrent occurrences in different sets (right column). The numbers on the bottom line summarize the total number of recurrent genes for each set.

Approximately half of the top genes in each set occur in other sets, 31 in a single other set, the others in 3 to 7 other sets (Fig. 4). Among the two that occur in the largest number of other sets (seven) is the zinc-finger transcription factor SALL3, which has been implicated in cancer cell development in humans (Álvarez et al. 2021). It is among the most strongly down-regulated among all transcripts. The other gene that occurs in seven sets is a novel transcript (ENSG00000269547) without known function. Interestingly, this gene shows a positive log2-fold change in 6 sets, but a negative in one set (Mdng3). Such opposite regulatory effects are also found for 7 other genes that occur in more than one set (marked with framed boxes in Fig. 4).

### Structural Features of Mdng ORFs

To assess the structural features of the Mdng ORFs, we calculated the average intrinsic disorder score (IDS) for each Mdng, based on flDPnn (Hu et al. 2021) (Table 2). Based on IDS > 0.4, we classify the foldability as low, and for IDS < 0.1 as high (Table 2). The IDS score does not correlate significantly with the rank order of relative growth in the common growth environment experiment (Spearman rho = −0.07, *P* [2-tailed] = 0.8), i.e. it is not a predictor of possible function in the cell.

**Table 2:** Structural feature of MDNG ORFs

	Protein length	IDS (flDPnn)	Foldability	Rank in common growth experiment
Mdng1	147	0.51	Low	2
Mdng2	167	0.48	Low	n.a.
Mdng3	157	0.08	High	5
Mdng4	122	0.06	High	4
Mdng5	111	0.43	Low	7
Mdng6	48	0.50	Low	10
Mdng7	143	0.19	…	n.a.
Mdng9	133	0.23	…	6
Mdng10	155	0.17	…	11
Mdng11	91	0.06	High	12
Mdng12	155	0.24	…	9
Mdng13	101	0.11	…	14
Mdng14	101	0.63	Low	13
Mdng15	102	0.28	…	1
Mdng16	70	0.18	…	3
Mdng21	115	0.09	High	8

**Fig. 5.**
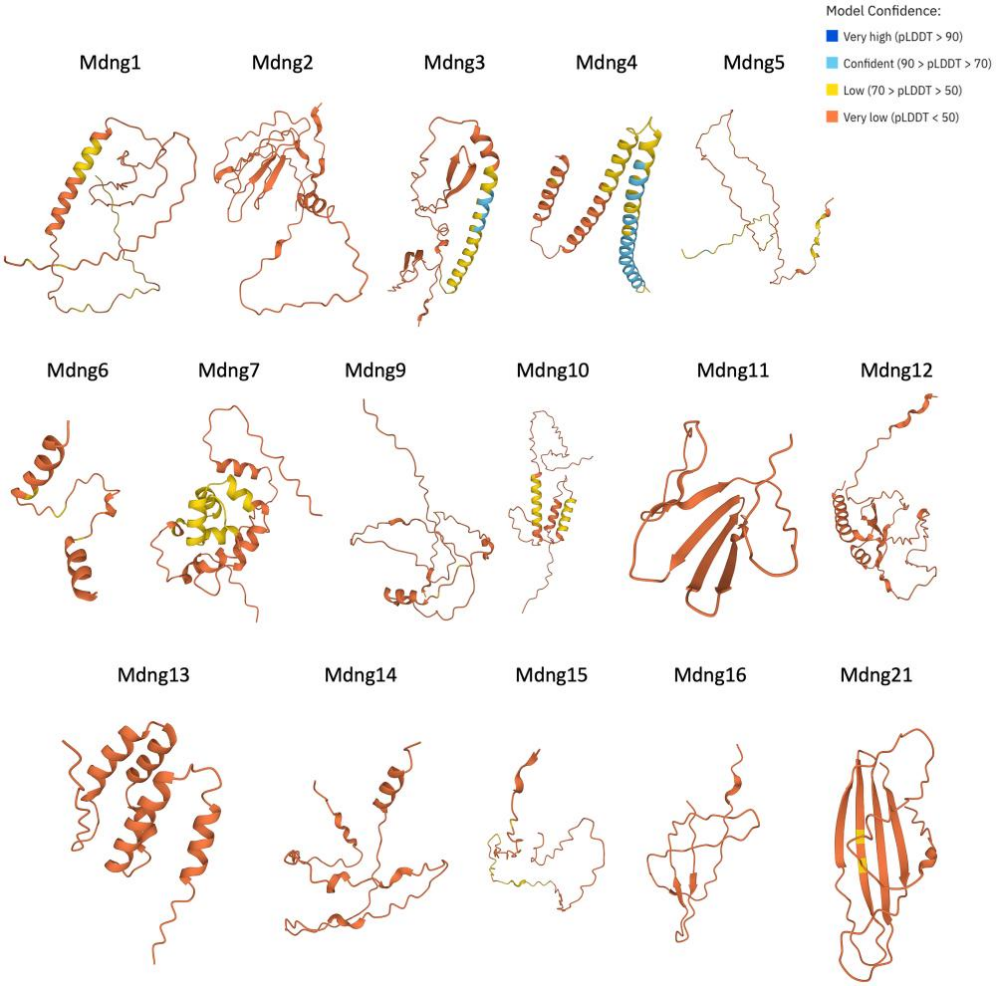
AlphaFold predictions for Mdng genes in the study. Individual structure predictions were downloaded from the AlphaFold ProteinStructure Database (https://alphafold.com/). See text for further details.

Second, we checked the AlphaFold predictions of the Mdng genes in the experiment, which are part of all mouse gene predictions (Jumper et al. 2021; Varadi et al. 2022). The predicted structures are displayed in Fig. 5. Consistent with the fact that AlphaFold significantly relies on sequence similarity and its accuracy is low (∼35%) for ab initio structure prediction (Jumper et al. 2021), the predictions for Mdng proteins do not have very high confidence (indicated by the yellow and red colors). Most predictions are alpha-helices, while two proteins show major beta-sheet parts (Mdng11 and Mdng21). For comparison, we have also used RaptorX (Källberg et al. 2012; Wang et al. 2016) for predicting structural elements along the sequences of all Mdng genes (supplementary table S6, Supplementary Material online). Most agree with the AlphaFold predictions, with only minor differences, except for Mdng11, where RaptorX predicts more alpha-helical parts than AlphaFold. Note, however, given the high uncertainty of these predictions, they would likely have to be revised if a full structural analysis of the proteins is conducted. Nonetheless, we show the predictions here, since they are useful for indicating that different structural elements can potentially form in the de novo evolved genes, although the ones shown may not be the finally correct ones.

## Discussion

Our study includes two experimental series to tackle the impact of expression of proteins derived from random sequences in human cells. In the first we applied competitive growth experiments with a random sequence library, analogous to previous experiments conducted in *E. coli* (Neme et al. 2017; Castro and Tautz 2021), but with expression from a defined chromosomal site, rather than from an extrachromosomal plasmid. In the second, we used cDNAs identified as de novo genes in the mouse lineage and expressed them in human cells. These genes have emerged out of noncoding sequences that can be considered to be more or less random, although not in the sense of true randomness as for the synthetic random sequences since eukaryotic sequences are also shaped by duplication and slippage processes that make them internally more repetitive (Tautz et al. 1986; Karlin and Mrázek 1997). Further, the Mdngs tested here are expected to be pre-selected for being compatible with expression in a mammalian cell background.

For the random sequences, we found differential fitness effects when grown together in a pool, with a substantial proportion of clones either declining or increasing in frequency over time. Interestingly, the distribution of molecular features such as length and aggregation propensity did not correlate with the observed fitness outcomes, which is different from what was found for fitness effects of random sequences in *E. coli* (Castro and Tautz 2021; Kosinski et al. 2022). In contrast to the bacterial experiments, the experiments with human cell lines are not direct competition experiments, since the cells grow individually attached to a surface and are re-passaged for the next growth cycle before they reached confluence. Hence, the enrichment or decline of clones expressing a certain peptide or Mdng must be due to effects on the general growth properties of the cells under common conditions.

The positive and negative growth effects of the clones can be seen as relative to each other in the given experiment. This does not imply that they would confer a fitness advantage for the whole organism. Still, one can conclude that random peptide expression in eukaryotic cells can have differential effects on cell physiology. The same overall conclusion can be derived from the experimental series with the Mdng clones. Each has a differential effect on the cells in which they are expressed and they can lead to differences in growth potential.

The transcriptomic analysis provided further insights into the cellular responses elicited by the expression of the Mdngs. The observed variations in the expression levels of human genes in response to different Mdngs suggest that each Mdng exerts a unique impact on the cellular transcriptome. The enrichment of specific Gene Ontology (GO) terms associated with different Mdngs further underlines their functional specificity. Notably, the upregulation of genes involved in processes such as cell adhesion, ion transport, and biosynthetic pathways suggests that Mdngs may modulate diverse cellular functions. Of special interest is also the identification of many human-specific transcripts among the top differentially regulated genes, implying that human de novo genes can be directly or indirectly regulated by new de novo genes, such as the Mdngs.

The assessment of structural features and predicted protein structures of Mdng ORFs provides no conclusive insights into how they might function. The observed range of intrinsic disorder scores and foldability suggests a diversity of structural properties among Mdngs, as expected based on their origin from largely random sequences. Only for Mdng11, we find an interesting pattern, with a predicted localization at the cell surface and a significant enrichment in the GO term for genes involved in homophilic cell adhesion.

The absence of a significant correlation between structural features and growth outcomes suggests that a predictability on possible adaptive functions cannot be derived from the sequence features alone, at least for this limited set of cases. In any case, while structure predictors can provide initial insights into the structural composition of de novo and random proteins (Liu et al. 2023) their accuracy and applicability to such proteins remain limited (Aubel et al. 2023; Middendorf and Eicholt 2024).

Note that we cannot distinguish in our experiments between the effects exerted by the expression of the Mdngs as a consequence of the expressed RNA or of their translation products. *De novo* evolved genes in mouse may also act as noncoding RNAs (e.g. [Heinen et al. 2009]) and there is also evidence from expressing random sequences that their RNA component may contribute to the effects (Castro and Tautz 2021).

## Conclusion

Our results from expressing random sequences and mouse de novo genes show that a large fraction of them can trigger changes in the growth of the cells. For the mouse de novo genes we also showed that each triggers a different overall transcriptomic response in the heterologous human cells, in which they were never expressed before. These observations strengthen the notion that de novo genes could directly become functional when the selection conditions allow this. They can potentially work at the time where they are first expressed and do not necessarily need further evolutionary optimization to be functional, since already their initial sequence can exert a biological effect. Some may already form stable folds from scratch, as it is predicted by the structure analysis programs. Interestingly, a comparative analysis of de novo evolved genes in *Drosophila* lineages with ancestral sequence reconstruction has shown that most potentially well-folded candidates are already born well-folded (Peng and Zhao 2024).

## Materials and Methods

### Library of Random Sequences for Eukaryotic Cells

The random sequence library was designed to have 150 randomly synthesized nucleotides flanked by fixed features. On the 5′ end of the random region, all sequences have a HindIII restriction site for cloning and a Kozak sequence including a start codon for transcription and translation initiation. On the 3′ end of the random region, a histidine tag (6xHis-Tag) for protein detection, a stop codon, and a NotI restriction site for cloning were included. In order to increase the probability of successful expression of the tags in a human cell line, care was taken to use histidine codons with a mid- to high-frequency of use in human genes. The full length of the ORF designed in the library is 174 bp (58 aa), including the 150 bp (50 aa) random region (supplementary file S1, Supplementary Material online).

The oligonucleotide pool for the library was ordered from metabion GmbH as a single-stranded oligonucleotide and amplified using specific primers. To avoid enrichment of partial sequences or PCR errors, only the reverse primer was added for an initial long amplification cycle with 30 s annealing at 55 °C and 20 min extension at 72 °C, following the addition of the forward primer another long amplification cycle with annealing temperature of 60 °C was followed by six two-step amplification cycles: 98 °C for 10 s, and 72 °C for 1 min. Products with the right size were purified from an agarose gel, cut using HindIII-HF (NEB, Cat. No. R3104S) and NotI- HF (NEB, Cat. No. R3189S), and ligated into a pcDNA5/FRT/TO plasmid. Ligated plasmids were cloned into chemo-competent *E. coli* JM109 (Promega, Cat. No. L1001) for amplification.

### Cell Lines and Cell Culture Conditions

Flp-In T-REx 293 cells (Invitrogen, Cat No. R780-07) were cultured in complete medium (DMEM (Gibco, Cat No. 41966-029), 10% FBS Tet-free (PAN Biotech, Cat No. P30-3602)) with the required antibiotics: 15 µg/mL blasticidin S HCl (Gibco, Cat No. A1113903), 100 µg/mL Zeocin (Invitrogen, Cat No. R25001), and/or 100 µg/mL hygromycin B (Invitrogen, Cat No. 10687010)). Cells were grown in a humidified incubator at 37 °C with 5% CO_2_. Cell counting was performed with Countess II FL Automated Cell Counter.

### Transfection and Selection

For transfection, 5 × 10^4^ Flp-In T-REx 293 cells were seeded in each well of a 24-well plate, with 1 mL complete medium plus blasticidin and zeocin and incubated for 2 d. The medium was changed to a complete medium without antibiotics 1.5 h before transfection with FuGENE 6 Transfection Reagent (Promega, Cat No. E2693). The supplier's protocol was followed using a FuGENE:(total)DNA ratio of 3:1, with vectors pcDNA5/FRT/TO/and pOG44 (Invitrogen, Cat No. V6005-20) in a ratio of 1:9, for a final volume of 25 µL transfection mixture. Opti-MEM I Reduced Serum Medium (Gibco, Cat No. 31985047) and FuGENE 6 were first incubated 5 min at room temperature, DNA was added and incubated for 15 min at room temperature, and then the mixture was added to the wells. Cells were kept at 37 °C. Twenty-four hours after transfection, the medium was changed to complete medium with blasticidin. Forty-eight hours after transfection, cells were resuspended in complete medium with blasticidin and hygromycin and split 1:4, transferring all cells from each well of the 24-well plate into wells of 6-well plates. The medium was refreshed by replacing 50% to 75% of the old medium with new one every 3 to 4 d. After 11 d, all cells in negative controls were dead and every candidate had growing colonies of selected cells.

For each candidate, all cells in the well were transferred to a T-75 flask, keeping the cells in complete medium with blasticidin and hygromycin. Cells were passaged at ∼80% confluency.

### DNA and RNA Extraction From Cells

For DNA extraction, cells at ∼80% confluency were washed, trypsinized and 1 mL single-cell suspension was centrifuged at 300 g for 5 min, at room temperature. Medium was removed and samples were stored at −20 °C before applying the DNeasy Blood & Tissue Kit (QIAGEN, Cat No. 69504) protocol for DNA extraction, with final resuspension in 200 µL Buffer AE. DNA was quantified with Nanodrop to assess concentration and quality.

For RNA extraction, cells at ∼80% confluency were washed, trypsinized, and suspended in complete medium with blasticidin and hygromycin. 2 × 10^5^ cells were seeded in 24-well plates, into 800 µL complete medium with blasticidin, hygromycin, and 50 ng/mL doxycycline. Cells were incubated at 37 °C for 24 h. The medium was removed and 250 µL TRIzol (Life Technologies, Cat No. 15596026) was immediately added and pipetted to homogenize the mixture. Samples were left at least 5 min at room temperature for incubation, or frozen at −20 °C. RNA isolation was done with the Direct-zol RNA Miniprep Plus Kit (Zymo Research, Cat No. R2070), including DNase I digestion. Samples were quantified with Nanodrop to assess RNA quantity and quality. RevertAid First Strand cDNA Synthesis Kit (Thermo Scientific, Cat No. K1621) was used for retrotranscription, using the manufacturer's protocol for total RNA (300 to 600 ng) with Oligo (dT)18 primer.

### Amplicon Sequencing for Library Experiments

Amplicon sequencing primers were designed for two-step PCR-based sequencing. The first PCR for the amplicon sequencing was performed in triplicate, using Q5 Hot Start High-Fidelity 2 × Master Mix (Cat. No. M0494S, NEB), with the following cycling program: initial denaturation at 98 °C for 30 s, followed by 24 cycles of 98 °C for 10 s, 62 °C for 30 s, and 72 °C for 20 s, and a final elongation cycle at 72 °C for 2 min. PCR products were pooled, product size was confirmed on an agarose gel and purified using double-sided selection with SPRIselect (Beckmann- Coulter, Cat. No. B23318). The second PCR was done with indexed Illumina primers, and the following cycling conditions: initial denaturation at 98 °C for 30 s, followed by 5 cycles of 98 °C for 10 s, 60 °C for 30 s, and 72 °C for 20 s, with a final elongation cycle at 72 °C for 2 min. The products were purified using AmpureXP beads (Beckmann- Coulter, Cat. No. A63881). Each barcoded sample was quantified and then pooled together in equal concentrations to obtain the sequencing library. DNA concentration was quantified with an Agilent Bioanalyzer using Agilent DNA7500 kit and with a NanoDrop 3300 using Qubit ds DNA BR Assay Kit.

All samples were sequenced in one Illumina NextSeq run. Samples had on average 696,779 paired-end reads. On average, 86% of clean forward reads were successfully mapped to the database. Count tables for each time point were compared to the first time point using DESeq2. The generation of the reference library of sequences was done as described in (Castro and Tautz 2021).

### Eukaryotic Library Time Course Experiment

The experiment was done starting from a frozen aliquot of the library with 3.5 million cells. The aliquot was thawed and seeded in a T75 flask with 14 mL of medium. After 80% confluence was reached, cells were passaged into two T150 flasks with 20 mL of medium, plus antibiotics (blasticidin and hygromycin) and allowed to grow again to at least 70% confluence. Cells were trypsinized and collected in a single tube and used to seed five T75 flasks with 3 × 10^6^ cells each. Doxycycline was added to a final concentration of 10 ng/mL at the seeding stage. At each subsequent passage every two days, one-fourth of the cells of each flask was seeded into a new one, and the remaining cells were stored for later DNA extraction. To do this, aliquots were centrifuged, the supernatant was removed, and pellets were flash-frozen in liquid nitrogen. Sampling was done for 10 time points.

### Sequence Feature Determination for Random Sequences

Sequence length was calculated for each read, as well as the predicted ORF and peptide encoded by them using bash programs during the database generation. The number of peptides of each length depends on the probability of getting a stop codon at each consecutive position, and not before. This is best described by the probability function of a geometric distribution (1–*P*)^(k-1)*P^, where *k* is the number of trials, in this case, the number of positions or the length of the sequence; and *P* is the probability of “success” getting a stop codon. Multiplying this probability distribution by the number of synthesized sequences, we obtained the expected count of peptides of each length. In addition to this, it is possible to predict the possible number of unique sequences of each length using the exponential function 20*^k^*, to describe the number of possible unique combinations of the 20 amino acids in a sequence of length *k*.

GC content was calculated for full reads and predicted ORFs using custom Perl scripts as the percentage of guanine (G) and cytosine (C) in a sequence relative to its length. Amino acid composition of the database and different sequence groups were calculated using the Biostrings package from Bioconductor in R (https://bioconductor.org/).

Protein aggregation propensity was calculated for each sequence using the program PASTA 2.0 (Walsh et al. 2014) on the web server of The BioComputing UP lab of the University of Padua (Italy). For each sequence, free energy for the best pairing was obtained using the default settings for peptides. The best energy pairing for self-aggregation was obtained for each sequence, and energies of −5 or less were considered indicative of a high probability of aggregation.

### Candidate Mouse de Novo Genes (Mdng)

Candidates were chosen from a previous list of candidate de novo genes, based on datasets for RNA-seq, Ribo-seq, and proteomic analyses (Xie et al. 2019). They were renamed MDNGX (Mouse De Novo Gene), where “X” is the candidate's number (supplementary table S3, Supplementary Material online).

Gene age was estimated by phylostratigraphy, whose central idea is estimating the minimal evolutionary birthdate of a protein-coding gene in a species by finding the most distant species in which a sufficiently similar protein sequence exists, and inferring that the gene was already present in the ancestor of the query species and the most distant species (Domazet-Lošo et al. 2007). Here this entails using a sequence similarity algorithm, BLASTP (Altschul et al. 1990), with the protein sequence of each Mdng mouse gene to search a large and taxonomically well-covered database and find for each Mdng protein the most distant similar protein with an e-value threshold of better than 10^−3^. The minimal length of the protein for which gene age can reliably be estimated is 40 amino acids (Weber et al. 2020; Fleck et al. 2024). While the NCBI nonredundant database is widely used (Weber et al. 2020), here we used a smaller database which we built from UniProt 2020 and slimmed down using a taxonomically informed pruning algorithm that reduces database size and increases search speed by preferentially removing species in well-covered taxonomic neighborhoods (Arendsee et al. 2019). Separately, we repeated the searches with the NCBI nr database (February 2024). The age of each gene is estimated as the TimeTree (https://timetree.org/, [Kumar et al. 2022]) evolutionary age, measured in million years ago (Mya).

Based on these results, as well as population re-sequencing data (Harr et al. 2016), we assigned the most distant species in which a credible orthologous ORF could be found (listed in supplementary table S3, Supplementary Material online).

### Mdng Protein Sequence Analysis

The presence of features determining protein subcellular localization, such as signal peptides (SP) and transmembrane domains (TMD), was scored using secondary structure prediction algorithms TargetP, HMMER, Phobius and PredictProtein (Käll et al. 2007; Almagro Armenteros et al. 2019; El-Gebali et al. 2019; Bernhofer et al. 2021). Protein sequence alignments were performed pairwise in BLASTP (Altschul et al. 1990) and with multiple sequences in MAFFT (Rozewicki et al. 2019), using the accuracy-oriented MAFFT L-INS-i option, and employing JalView (Troshin et al. 2018) for visualization and data storage. Intrinsic disorder analysis was done with flDPnn (Hu et al. 2021).

For overall structural analyses, we used the AlphaFold ProteinStructure Database (https://alphafold.com/). The AlphaFold structure predictions generally had low confidence, likely because of the lack of related proteins with known structures. The algorithm for the RaptorX website (Källberg et al. 2012; Wang et al. 2016) was used as an alternate source of structural prediction. While it performs more accurately with profiles, it can also run on raw sequences. v1.10, 2018.10.01 of Predict_Property (a command-line version) was downloaded from https://github.com/realbigws/Predict_Property and run with the default parameters on each of the 16 Mdng proteins.

### Cloning of Mdng cDNAs for Expression Constructs

Gene-specific primers were designed on the CDS (supplementary table S7, Supplementary Material online) and used to amplify the genes either from genomic DNA (C57Bl6) or cDNA (derived from testis RNA). PCR reactions to amplify the candidates from gDNA/cDNA were performed with Phusion High-Fidelity PCR Master Mix with HF Buffer (Thermo Scientific, Cat No. F531). The Melting temperature (*T*_m_) for each primer pair was calculated using Thermo Fisher online Tm calculator. The first amplification was done with gene-specific primers, using actin as a reference gene for gDNA amplification and TBP as a reference gene for cDNA amplification. The second amplification was done with gene-specific primers with restriction enzyme extensions.

PCR products that showed a single band on the gel were purified either with QIAquick PCR Purification Kit (Cat No. 28104), or SPRIselect beads (Beckman Coulter, Cat No. B23318). PCR products that showed extra bands on the gel were purified from the gel with the QIAquick Gel Extraction Kit (Cat No. 28704).

The inserts obtained after the second amplification were ligated into the pcDNA5/FRT/TO vector (Invitrogen, Cat No. V652020) using the corresponding double digests for each clone. After ligation, they were transformed into *E. coli* strain JM109 Competent Cells (Promega, Cat No. L1001). Successful cloning was checked via Sanger sequencing of each clone used for the further steps.

### RNA-Seq and Data Analysis

RNA was sequenced with Illumina NovaSeq based on the TruSeq Stranded mRNA Sample Preparation Guide (Rev. D September 2012). FASTQ converted reads were trimmed with Trimmomatic (0.39) (Bolger et al. 2014). Only paired-end reads left were used for the following analyses. Trimmed reads were mapped to the human genome (GRCh38) with HISAT2 (2.2.1) (Kim et al. 2019) and Samtools (1.15.1) (Li et al. 2009); the human gene annotations were obtained from Ensembl (Version 105), while the mouse genes and the hygromycin resistance encoded by the modified Flp-In T-REx cell lines were manually added to the reference file. We counted fragments mapped to the genes annotated by Ensembl (Version 105) (Howe et al. 2021) with Stringtie (2.2.1) (Pertea et al. 2015). For differential expression analysis, we used DESeq2 (1.36) (Love et al. 2014). Gene ontology analysis was done with GOrilla (Eden et al. 2009) using the “Two unranked lists of genes” option, with the set of expressed human genes as the background list. The results of these analysis are deposited as Excel workbook “all_DeSeq2_and_GO.xlsx” at Edmond V1 (https://doi.org/10.17617/3.BNGHVS)

### Mdng Common Growth Experiment

A frozen aliquot of each Mdng Flp-In T-REx cell line was thawed, together with a GFP cell line. For each T-150 flask, four Mdng cell lines were inoculated into 35 mL of medium—except for one, containing four Mdng + GFP control. After 80% confluence was reached, for the first and second passage (1:4) all cell lines were pooled together into a single T-150 flask, with medium plus blasticidin/hygromycin. Cells were trypsinized and collected in a single tube, counted, and used to seed five T-25 flasks with 7 × 10^5^ cells each. Doxycycline was added to a final concentration of 50 ng/mL at the seeding stage. At each subsequent passage every two days, one-fourth of the cells of each flask was seeded into a new one, and the remaining cells were used for later DNA extraction. Aliquots were centrifuged at 300×g for 5 min, supernatant was removed, and pellets were flash-frozen in dry ice. Sampling was done for five time points, plus the initial seeding (T0).

## Supplementary Material

evae175_Supplementary_Data

## Data Availability

All relevant data are provided in the supplementary tables. The sequence read files were submitted to the European Nucleotide Archive (ENA) under the project number PRJEB74506. An excel file with all individual DESeq2 and GO results for the Mdng genes is deposited at: https://doi.org/10.17617/3.BNGHVS.
