# Supplementary material for: Expression of Random Sequences and de novo Evolved Genes From the Mouse in Human Cells Reveals Functional Diversity and Specificity
Source: Genome Biol Evol. 2024 Dec 12;16(12):evae175. doi: 10.1093/gbe/evae175 (PMC11635099; doi:10.1093/gbe/evae175)
Supplement: evae175_Supplementary_Data [file evae175_supplementary_data.zip › Supplementary_files_all.docx]

**List of Supplementary files**

**Supplementary file 1 (pages 1-6)**

Construction and features of the random sequence library

*Suppl. Figure 1.1. Experimental design for the random sequence library.*

*Suppl. Figure 1.2. Flp-In™ T-REx™ system.*

*Suppl. Figure 1.3. Distribution of predicted peptide lengths in the library.*

*Suppl. Figure 1.4. GC content of sequences in the library of random sequences.*

*Suppl. Figure 1.5. Intrinsic disorder of predicted peptides in the database, binned by length.*

*Suppl. Figure 1.6. Distribution of aggregation energies for predicted peptides in the database.*

**Supplementary file 2 (pages 7-11)**

Results of the searches for remote homologues of Mdng genes, focused on Mdng12, Mdng14, Mdng15, for which matches were found outside the genus Mus.

**Supplementary tables in a single Excel file with 6 tabs**

**Supplementary Table S1**

IDs and associated information for all clones that were traced in the random sequence library

**Supplementary Table S2**

read counts for all random sequence clones from the common growth experiment

**Supplementary Table S3**

list of all Mdng sequences in the study

**Supplementary Table S4**

read counts for all Mdng sequence clones from the common growth experiment

**Supplementary Table S5**

sets of top 20 changed human genes for each Mndg expressed in the cells

**Supplementary Table S6**

summary results of RaptorX structure predictions for all Mdngs

**Supplementary Table S7**

primer sequences used in the study

**Supplementary file 1**

**Construction and features of the random sequence library**

The experimental system used for this study is a library of random sequences expressed in the human cell line HEK293. Each cell in the library is modified to express a single 174 nucleotide long sequence with 150 random nucleotides flanked by two constant codons at the 5’ end, and a 6-histidine tag at the 3’ end (suppl. Figure 1.1). Each random sequence acts as a barcode, and this makes it possible to quantify the relative number of cells in the population expressing it using an amplicon sequencing approach. In this way, one can monitor how the proportion of individual sequences changes over time under competitive growth conditions.


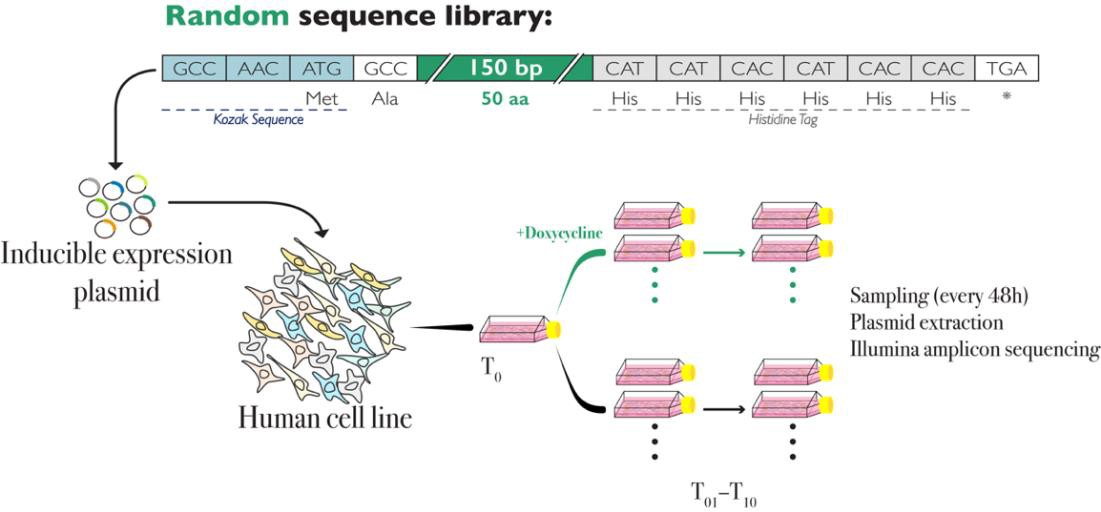


**Suppl. Figure 1.1. Experimental design for the random sequence library.**

The constant sequence flanking the random part of the oligonucleotides are depicted, including the Kozak sequence before the start codon and the 6 x histidine tag before the stop codon.

The library of random sequences was cultured with doxycycline to induce expression of the peptides over a period of 20 days, sampled every 48 hours and sequenced to determine the proportion of cells containing each sequence. The experimental design is similar to that used before in *E. coli* (Neme, et al. 2017), but with eukaryote specific genomic features in the flanking regions. Specifically, we used a targeted genome integration for the expression construct, a Kozak sequence to facilitate translation, shorter leading sequences on the 5’-end of the random sequence, and codon-optimized flanking sequences on both sides of the random sequence using frequently used codons in the human genome.

We used the Flp-In™ T-REx™ 293 cell line (FITR293, ThermoFisher Scientific). Flp-In™ T-REx™ is a protein expression system, selected for this study because of three key features: it permits the generation of stable expression cell lines; the expression construct is integrated into a specific target site on the genome; and expression is inducible with a Tet-On system (Figure 2-2). The FITR293 was generated by inserting two plasmids into the genome of the commonly used HEK293 cell line: the first one—pcDNA™6/TR—stably expresses the tetracycline repressor gene (tetR) and a Blasticidin resistance gene; the second one—pFRT/lacZeo—contains a Zeocin resistance gene with an FRT recombination site inside.


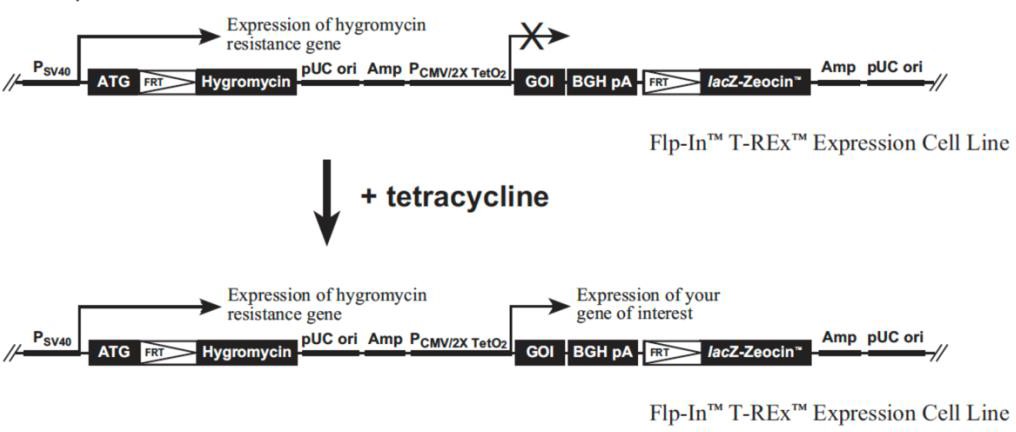


**Suppl. Figure 1.2. Flp-In™ T-REx™ system.** Figure reproduced from the provider’s Core Kit manual.

For the generation of the library, the oligonucleotides with the random sequences were inserted in the multiple-cloning site of a third plasmid—pcDNA5/FRT/TO. It contains a strong CMV promoter with two tetracycline operator (TetO_2_) sequences, an FRT site for targeted recombination with the one already on the genome, and a Hygromycin B resistance gene (*hygB*) without a start codon. This plasmid was co-transfected with a pOG44 plasmid, which contains a FLP integrase gene. The integrase, expressed transiently in the cells after transfection, mediates DNA recombination between the FRT site already on the genome, and the one in the plasmid with the insert to be expressed. Successful recombination resulted in the *hygB* gene gaining a start codon from the pFRT/lacZeo plasmid, which gave the cells Hygromycin resistance for selection of successful clones.


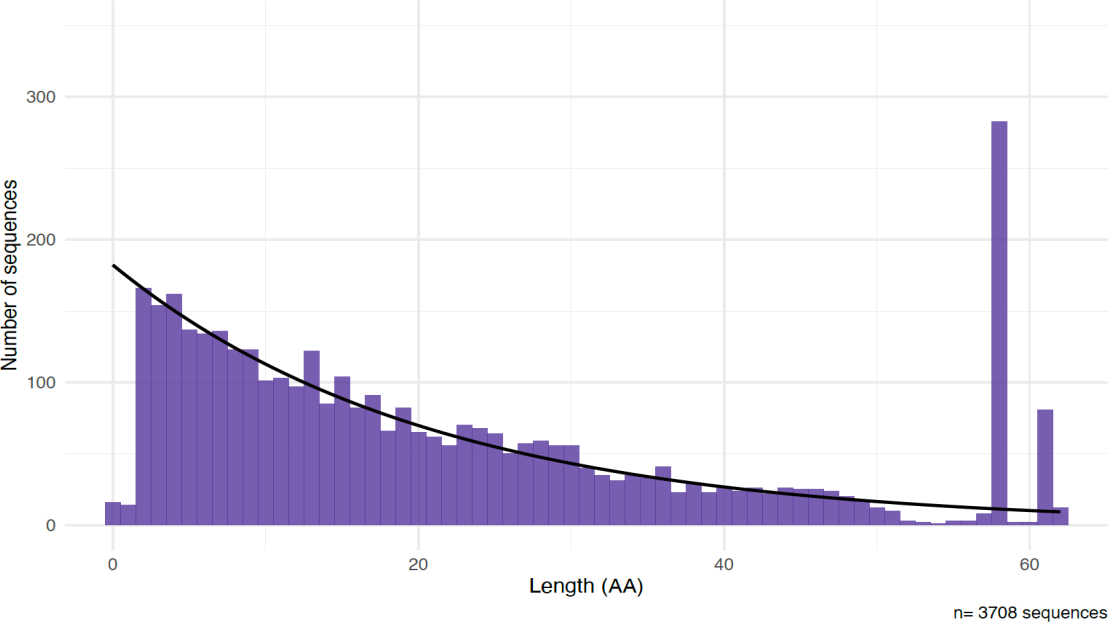


**Suppl. Figure 1.3. Distribution of predicted peptide lengths in the library.**

The black line corresponds to the probability mass function of a geometric distribution with p=3/64, considering the probability of having a stop codon at each position without having one in any previous one in the sequence before. Note that the peaks at the end of the distribution are due to the design of the library plus mutational frameshift effects at the end of the sequence (see (Castro and Tautz 2021) for discussion of this effect).


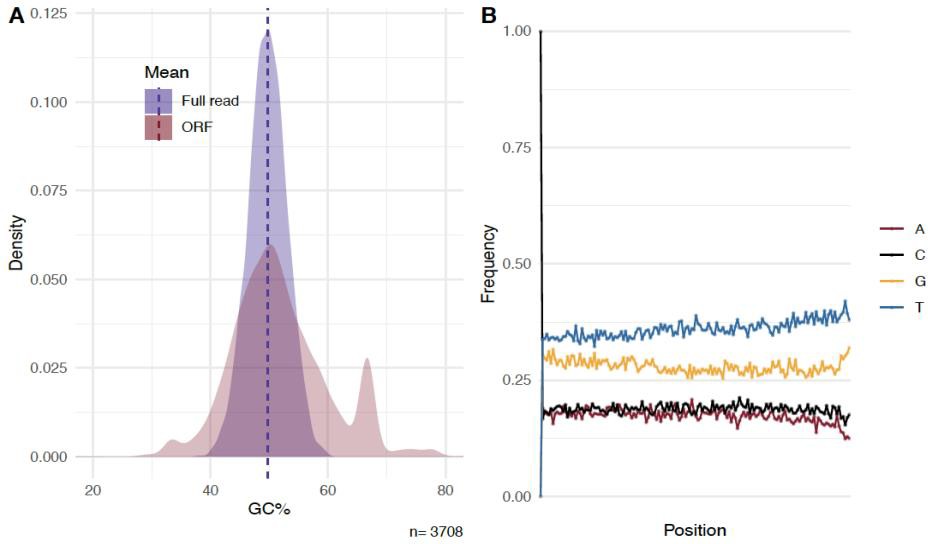


**Suppl. Figure 1.4. GC content of sequences in the library of random sequences.**

A. GC content distribution for full-length reads (blue, mean = 49.72%) and predicted ORFs in the database (red, mean = 51.94%). B. Average frequency of nucleotides at each position of the random part of the sequence (150 nucleotides). Note that there is a slight excess of G and T nucleotides. The total numbers of the respective nucleotides found across all sequences in the library (suppl. Table S1) are: 124782 A, 216948 T, 158747 C, 179209 G, which generates an overall of 337956 GC and 341731 AT nucleotides, i.e. a close to 50% GC content.

The main two features studied for the predicted peptides were intrinsic disorder and aggregation propensity. Intrinsic disorder was calculated as the average intrinsic disorder score of all residues in a sequence, calculated with the -short option of IUPred2A. Disorder scores are calculated based on features of each amino acid and their predicted interactions. Length is an important factor in these calculations and the software used cannot reliably assign disorder scores for peptides shorter than 30 residues. However, this analysis provides a good idea of the correlation between length and intrinsic disorder, and of GC content and intrinsic disorder.


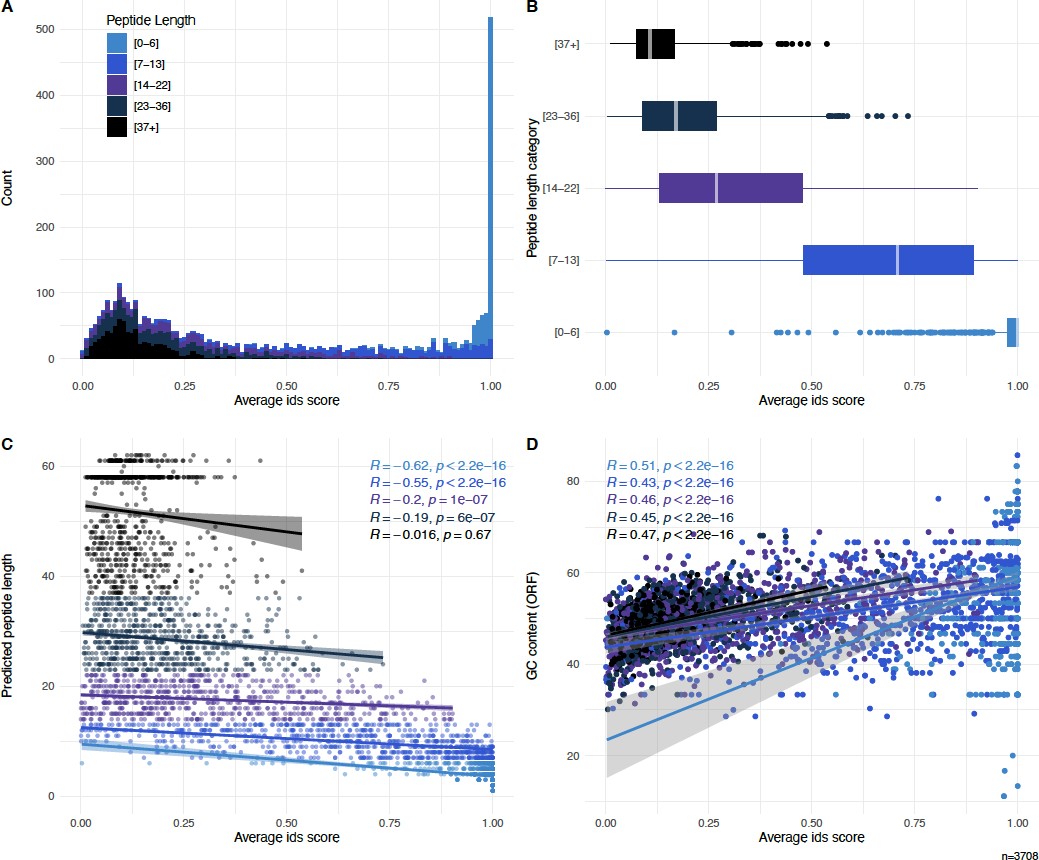


**Suppl. Figure 1.5. Intrinsic disorder of predicted peptides in the database, binned by length.**

A. Distribution of average intrinsic disorder scores. B. Boxplot of average intrinsic disorder scores for peptides in each length category. C. Correlation between peptide length and intrinsic disorder. D. Correlation between GC content and intrinsic disorder.

In the case of aggregation energy, a commonly used predictor of the likelihood that a sequence will aggregate in the cell and form amyloids, there is also a clear correlation with length. More sequences in the longest peptide length categories are predicted to be prone to aggregation.


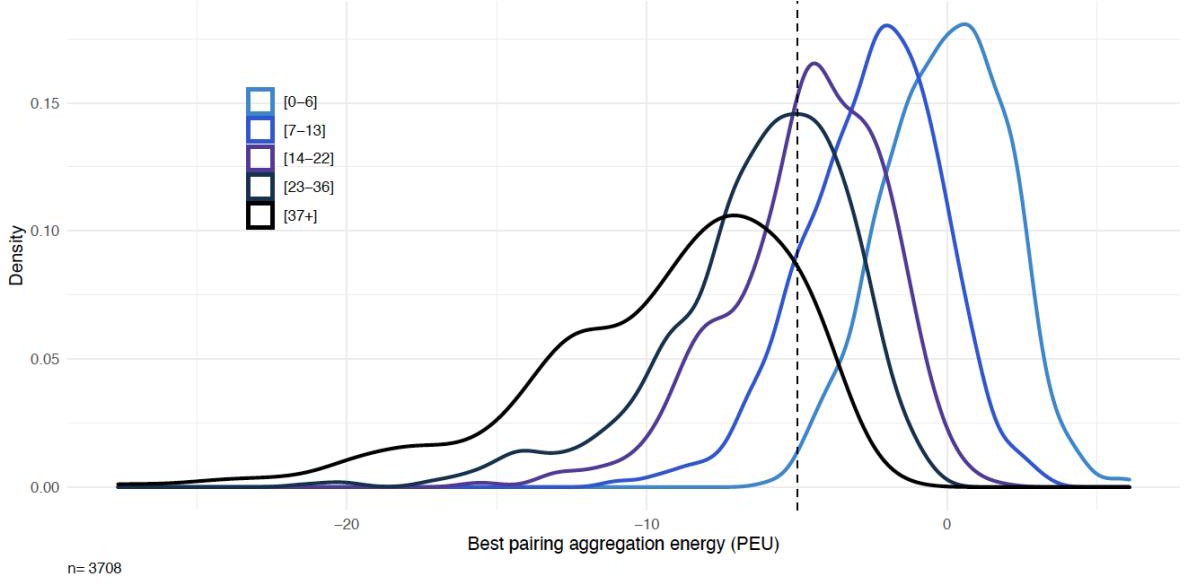


**Suppl. Figure 1.6. Distribution of aggregation energies for predicted peptides in the database.**

Aggregation energies are calculated with the software PASTA2.0, and expressed as "pasta aggregation units" (PEU). Peptides with PEU of -5 or lower are considered to be prone to aggregation.

Castro JF, Tautz D. 2021. The effects of sequence length and composition of random sequence peptides on the growth of E. coli cells. Genes 12.

Neme R, Amador C, Yildirim B, McConnell E, Tautz D. 2017. Random sequences are an abundant source of bioactive RNAs or peptides. Nature Ecology & Evolution 1.

**Supplementary file 2**

Results of the searches for remote homologues of Mdng genes, focused on Mdng12, Mdng14, Mdng15, for which matches were found outside the genus Mus.

Aligning sequences between these 3 *Muridae* Mdng proteins and the respectively similar *Cricetidae* proteins shows that half or more of the Mdng protein sequences are contained within alignments having 45% - 60% amino acid sequence identity (alignment lengths and sequence identity: Mdng12, *Mm* and *Pm*: 134 amino acids (aa), 49%; Mdng14 *Mm* and *Pm*: 88 aa, 56%, *Mm* and *Pr*, 93 aa, 60%; Mdng15, *Mm* and *Pr*: 73 aa, 47%). Aligning 3 or more sequences shows blocks of conservation of 30-40 aa with >50% of positions conserved (Mdng14, 32 aa, 56% conserved in 3 species; Mdng 15, 32 aa, 50% conserved in 5 species).

**Sequences similar to Mdng12, Mdng14, Mdng15.**

(i) 1 sequence for Mdng12

>Mm_Mdng12 (Mus musculus)

MPPLKPSRQISAGFRVSPKASIWDASGICSPSGPDQEAPALQGATAQSSGLGWKGGRHRLRVRSPDFGEKERKKCTLGKGASTFCACAFMEVTSSRLLKMLLRSAPSQAVAIRAKSSCPGRRALWVAPRHTIATTVRRYARYAYLLAKIKCKIKI

>Pm_ProtD_T0034921 (Peromyscus maniculatus)

MLPLKPSRQMSAGFRVYPQGKHIWGVSRQMLALGADQEAAALRGTTAQTRGPGGRVGGTAPRGMLSRLRRGRKEEMHPRKNGLRLFVPVLSWKSPALRLIKMLLRSAPSQAVASLAKSACPGRRPLRVTPGKKCKIKF

(ii) 2 sequences for Mdng14

>Mm_Mdng14 (Mus musculus)

MEIISWALNRELSSLSIRHTVPSSTHLQSGTEGGLSFGQCPPPQSTHWESRKGGRLAEHPPARNPACAPGPFGSPQVLALQKDLPWPDSGNSVTWSYSGGN

>Pr_1600014C23Rik (Phodopus roborovskii)

MEIISWALNRELSSLSIKHPVLSSTHPQTGTEGGLSLGQHPLLILPTGSPGKGGGWQHPPARNPACAPDV

TSCPFFPAIPFTSPQVLGLAEEPARAIGALEDGTFGLHQSWSLSWPFMYVVERVLPICSVWGRGILHRIA

CLKPMKPGLDVLDCITWDSRNEIPLSSIPHNPLPQDAALLTLPA

>Pm_1600014C23Rik (Peromyscus maniculatus)

MEIISWALNRELSSLFSHQASCAQFDPSTDWDRRGPVSWPVPPPHPTHWESEERGWLAKHPPARNPACAPVPFRNPQGLALAEGLAQAVGTQGPRTQEGN

(iii) 5 sequences for Mdng15

>Mm_Mdng15 (Mus musculus)

MAQNHTDVELSAPSPEPCLPACHHVSRHDENGLNLQTKYRRRKRKPWKENAALPRLSIRNSAAVSTGFSRLLLLDAVSPGLMRLPFVCSLNLATPVVHDVCC

>XP_021075259.1 (Mus pahari)

MDESAKTLPPPRLCFCPEKGEDMKVGYDPIITPQKEEGAWVGICRDGRLLAATLLLALVSSSFTAMSLYQLAALQADLMSLRMELQSYRGSATPAAPSVPGLTAEVKLLTPAAPQPYNSSRGHRNRRASQGPGETEQDVELSAPPAPCLPGCRHSQHDDSGLNLRTRTYTFVPWLLSFKRGNALEEKENKIVVRQTGYFFIYSQVLYTDPIFAMGHVIQRKKVHVFGDELSLVTLFRCIQNMPKTLPNNSCYSAGIARLEEGDEIQLAIPRENAQISRNGDDTFFGALKLL

>XP_048297371.1 (Myodes glareolus)

MIIEALDVPWVVLLSMDSFYKVLTQQQQEQAACNNFNFDHPDAFDFDLIISTLKKLKQGRSVQIPIYDFTTHSRKKDWKTLYGANVIIFEGIMAFADKTLLELLDMKIFVDTDSDIRLVRRLRRDISERGRDIEGVIKQYNKFVKPAFDQYIQPTMRLADIVVPRGSGNTVAIDLIVQHVHSQLEERKLRWDMAALASAHQCHPLPQTLSVLKSTPQVRGMHTIIRDKETSRDEFIFYSKRLMRLLIEHALSFLPFQDCTVQTPQGQDYVGKCYAGKQITGVSILRAGETMEPALRAVCKDVRIGTILIQTNQLTGEPELHYLRLPKDISDDHVILMDCTVSTGAAAMMAVRVLLDHDVPEDKIFLLSLLMAEMGVHSVAYAFPRVRIITTAVDKRVNDLFRIIPGIGNFGDRYFGTDAVPDGSDEEETASVEQDVELSASSPAPCLPACCPVSRHDDNGLNF

>CAH7467545.1 (Phodopus roborovskii)

MKKPLSILNHLTKELSLKLLNHLYVDLSAPSPTPFLPACHHASCHDNNGLNLQTLGSLIMVRGVAQKNWVRDILNPKGHLKDLISKSESFSKL

>XP_038188112.1 (Arvicola amphibius)

MAAPPASADATPSSLQSAVAPDAPGRPAEQTETACEDRSNTGSLDRLLPPVGTGRSPRKRTTSQCKSEPPLLRTSKRTIYTAGRPPWYNEHGTQSKEAFAIGLGGGSASGKTTVARMIIEALDVPWVVLLSMDSFYKVLTQQQQEQAACNNFNFDHPDAFDFDLIISTLKKLKQGRSVQIPIYDFTTHSRKKDWKTLYGANVIIFEGIMAFADKTLLELLDMKIFVDTDSDIRLVRRLRRDISERGRDIEGVIKQYNKFVKPAFDQYIQPTMRLADIVVPRGSGNTVAIDLIVQHVHSQLEERELSVRAALASAHQCHPLPQTLSVLKSTPQVRGMHTIIRDKETSRDEFIFYSKRLMRLLIEHALSFLPFQDCTVQTPQGQDYVGKCYAGKQITGVSILRAGETMEPALRAVCKDVRIGTILIQTNQLTGEPELHYLRLPKDISDDHVILMDCTVSTGAAAMMAVRVLLDHDVPEDKIFLLSLLMAEMGVHSVAYAFPRVRIITTAVDKRVNDLFRIIPGIGNFGDRYFGTDAVPDGSDEEETASVEQDVELSASSPALCLPACCHVSRHDDKGLNLWL

>Pm_ProtD_T0050608 (Peromyscus maniculatus)

MCPKICVVFEISREDFILIYFCLLFMSSRADSFFGSRSFSAHLLECLGSGIRKRGLVARSLSWGWALRFHMLMPGQVSLSLPAACRSDVELSASSPAPCLPAHLHASCHDDNGLNF

**Pairwise sequence alignments in BLASTP between Mdng12, Mdng14 and Mdng15 and their respective best hits. The sequence of the best pairwise alignments are shown.**

(i) **Mdng12**

Mdng12 in *Mus musculus* and hit in *Peromyscus maniculatus*, alignment length 134 aa

| **Alignment statistics for match #1** | | | | | |
| --- | --- | --- | --- | --- | --- |
| Score | Expect | Method | Identities | Positives | Gaps |
| 89.0 bits(219) | 6e-28 | Compositional matrix adjust. | 66/134(49%) | 77/134(57%) | 10/134(7%) |

Query 1 MPPLKPSRQISAGFRVSPKA-SIWDASGICSPSGPDQEAPALQGATAQSSGLGWKGG--- 56

M PLKPSRQ+SAGFRV P+ IW S G DQEA AL+G TAQ+ G G + G

Sbjct 1 MLPLKPSRQMSAGFRVYPQGKHIWGVSRQMLALGADQEAAALRGTTAQTRGPGGRVGGTA 60

Query 57 -RHRL-RVRSPDFGEKERKKCTLGKGASTFCACAFMEVTSSRLLKMLLRSAPSQAVAIRA 114

R L R+R E +K G F + + RL+KMLLRSAPSQAVA A

Sbjct 61 PRGMLSRLRRGRKEEMHPRK----NGLRLFVPVLSWKSPALRLIKMLLRSAPSQAVASLA 116

Query 115 KSSCPGRRALWVAP 128

KS+CPGRR L V P

Sbjct 117 KSACPGRRPLRVTP 130

(ii) **Mdng14**

Mdng14 in *Mus musculus* and hit in *Peromyscus maniculatus*, alignment length 88 aa

| **Alignment statistics for match #1** | | | | | |
| --- | --- | --- | --- | --- | --- |
| Score | Expect | Method | Identities | Positives | Gaps |
| 79.0 bits(193) | 9e-26 | Compositional matrix adjust. | 49/88(56%) | 56/88(63%) | 7/88(7%) |

Query 1 MEIISWALNRELSSLSIRHTVPSSTHLQSGTE----GGLSFGQCPPPQSTHWESRKGGRL 56

MEIISWALNRELSSL S T+ G +S+ PPP THWES + G L

Sbjct 1 MEIISWALNRELSSLFSHQA--SCAQFDPSTDWDRRGPVSW-PVPPPHPTHWESEERGWL 57

Query 57 AEHPPARNPACAPGPFGSPQVLALQKDL 84

A+HPPARNPACAP PF +PQ LAL + L

Sbjct 58 AKHPPARNPACAPVPFRNPQGLALAEGL 85

Mdng14 in *Mus musculus* and hit in *Phodopus roborovski*i, alignment length 93 aa

**Alignment statistics for match #1**

|  | | | | | |
| --- | --- | --- | --- | --- | --- |
| Score | Expect | Method | Identities | Positives | Gaps |
| 88.6 bits(218) | 3e-28 | Compositional matrix adjust. | 56/93(60%) | 61/93(65%) | 11/93(11%) |

Query 1 MEIISWALNRELSSLSIRHTVPSSTHLQSGTEGGLSFGQCPPPQSTHWESRKGGRLAEHP 60

MEIISWALNRELSSLSI+H V SSTH Q+GTEGGLS GQ P KGG +HP

Sbjct 1 MEIISWALNRELSSLSIKHPVLSSTHPQTGTEGGLSLGQHPLLILPTGSPGKGGGW-QHP 59

Query 61 PARNPACAPG----------PFGSPQVLALQKD 83

PARNPACAP PF SPQVL L ++

Sbjct 60 PARNPACAPDVTSCPFFPAIPFTSPQVLGLAEE 92

(iii) **Mdng15**

Mdng15 in *Mus musculus* and hit in *Mus pahari*, alignment length 46 aa

| **Alignment statistics for match #1** | | | | | |
| --- | --- | --- | --- | --- | --- |
| Score | Expect | Method | Identities | Positives | Gaps |
| 47.0 bits(110) | 0.004 | Compositional matrix adjust. | 25/46(54%) | 30/46(65%) | 4/46(8%) |

Query 2 AQNHTDVELSAPSPEPCLPACHHVSRHDENGLNLQTKYRRRKRKPW 47

+ DVELSAP P PCLP C H S+HD++GLNL+T R PW

Sbjct 133 GETEQDVELSAP-PAPCLPGCRH-SQHDDSGLNLRT--RTYTFVPW 174

Mdng15 in *Mus musculus* and hit in *Myodes glareolus*, alignment length 34 aa

| **Alignment statistics for match #1** | | | | | |
| --- | --- | --- | --- | --- | --- |
| Score | Expect | Method | Identities | Positives | Gaps |
| 53.9 bits(128) | 2e-05 | Composition-based stats. | 24/34(71%) | 25/34(73%) | 0/34(0%) |

Query 2 AQNHTDVELSAPSPEPCLPACHHVSRHDENGLNL 35

A DVELSA SP PCLPAC VSRHD+NGLN

Sbjct 430 ASVEQDVELSASSPAPCLPACCPVSRHDDNGLNF 463

Mdng15 in Mus musculus and hit in *Phodopus roborovski*i, alignment length 73 aa

| **Alignment statistics for match #1** | | | | | |
| --- | --- | --- | --- | --- | --- |
| Score | Expect | Method | Identities | Positives | Gaps |
| 62.8 bits(151) | 4e-10 | Compositional matrix adjust. | 34/73(47%) | 43/73(58%) | 5/73(6%) |

Query 4 NHTDVELSAPSPEPCLPACHHVSRHDENGLNLQT-----KYRRRKRKPWKENAALPRLSI 58

NH V+LSAPSP P LPACHH S HD NGLNLQT R +K W + P+ +

Sbjct 21 NHLYVDLSAPSPTPFLPACHHASCHDNNGLNLQTLGSLIMVRGVAQKNWVRDILNPKGHL 80

Query 59 RNSAAVSTGFSRL 71

++ + S FS+L

Sbjct 81 KDLISKSESFSKL 93

Mdng15 in Mus musculus and hit in *Arvicola amphibius*, alignment length 34 aa

| **Alignment statistics for match #1** | | | | | |
| --- | --- | --- | --- | --- | --- |
| Score | Expect | Method | Identities | Positives | Gaps |
| 51.6 bits(122) | 1e-04 | Composition-based stats. | 24/34(71%) | 25/34(73%) | 0/34(0%) |

Query 2 AQNHTDVELSAPSPEPCLPACHHVSRHDENGLNL 35

A DVELSA SP CLPAC HVSRHD+ GLNL

Sbjct 545 ASVEQDVELSASSPALCLPACCHVSRHDDKGLNL 578

Mdng15 in *Mus musculus* and hit in *Phodopus roborovski*i, alignment length 73 aa

| **Alignment statistics for match #1** | | | | | |
| --- | --- | --- | --- | --- | --- |
| Score | Expect | Method | Identities | Positives | Gaps |
| 62.8 bits(151) | 4e-10 | Compositional matrix adjust. | 34/73(47%) | 43/73(58%) | 5/73(6%) |

Query 4 NHTDVELSAPSPEPCLPACHHVSRHDENGLNLQT-----KYRRRKRKPWKENAALPRLSI 58

NH V+LSAPSP P LPACHH S HD NGLNLQT R +K W + P+ +

Sbjct 21 NHLYVDLSAPSPTPFLPACHHASCHDNNGLNLQTLGSLIMVRGVAQKNWVRDILNPKGHL 80

Query 59 RNSAAVSTGFSRL 71

++ + S FS+L

Sbjct 81 KDLISKSESFSKL 93

Mdng15 in *Mus musculus* and hit in *Peromyscus maniculatus*, alignment length 34 aa

| **Alignment statistics for match #1** | | | | | |
| --- | --- | --- | --- | --- | --- |
| Score | Expect | Method | Identities | Positives | Gaps |
| 45.4 bits(106) | 2e-12 | Compositional matrix adjust. | 22/34(65%) | 24/34(70%) | 0/34(0%) |

Query 2 AQNHTDVELSAPSPEPCLPACHHVSRHDENGLNL 35

A +DVELSA SP PCLPA H S HD+NGLN

Sbjct 83 AACRSDVELSASSPAPCLPAHLHASCHDDNGLNF 116
